# Vision Transformers for Low-Quality Histopathological Images: A Case Study on Squamous Cell Carcinoma Margin Classification

**DOI:** 10.3390/diagnostics15030260

**Published:** 2025-01-23

**Authors:** So-yun Park, Gelan Ayana, Beshatu Debela Wako, Kwangcheol Casey Jeong, Soon-Do Yoon, Se-woon Choe

**Affiliations:** 1 Department of IT Convergence Engineering, Kumoh National Institute of Technology, Gumi 39253, Republic of Korea; alice3913@kumoh.ac.kr; 2 Department of Medical IT Convergence Engineering, Kumoh National Institute of Technology, Gumi 39253, Republic of Korea; gelan@kumoh.ac.kr; 3 School of Biomedical Engineering, Jimma Institute of Technology, Jimma University, Jimma 378, Ethiopia; 4 Center of Biomedical Engineering, Jimma University Medical Center, Jimma 378, Ethiopia; beshatudebela0@gmail.com; 5 Department of Animal Sciences, University of Florida, Gainesville, FL 32610, USA; kcjeong@ufl.edu; 6 Emerging Pathogens Institute, University of Florida, Gainesville, FL 32611, USA; 7 Department of Chemical and Biomolecular Engineering, Chonnam National University, Yeosu 59626, Republic of Korea

**Keywords:** squamous cell carcinoma, vision transformer, histopathology image, classification

## Abstract

**Background/Objectives:** Squamous cell carcinoma (SCC), a prevalent form of skin cancer, presents diagnostic challenges, particularly in resource-limited settings with a low-quality imaging infrastructure. The accurate classification of SCC margins is essential to guide effective surgical interventions and reduce recurrence rates. This study proposes a vision transformer (ViT)-based model to improve SCC margin classification by addressing the limitations of convolutional neural networks (CNNs) in analyzing low-quality histopathological images. **Methods:** This study introduced a transfer learning approach using a ViT architecture customized with additional flattening, batch normalization, and dense layers to enhance its capability for SCC margin classification. A performance evaluation was conducted using machine learning metrics averaged over five-fold cross-validation and comparisons were made with the leading CNN models. Ablation studies have explored the effects of architectural configuration on model performance. **Results:** The ViT-based model achieved superior SCC margin classification with 0.928 ± 0.027 accuracy and 0.927 ± 0.028 AUC, surpassing the highest performing CNN model, InceptionV3 (accuracy: 0.86 ± 0.049; AUC: 0.837 ± 0.029), demonstrating robustness of ViT over CNN for low-quality histopathological images. Ablation studies have reinforced the importance of tailored architectural configurations for enhancing diagnostic performance. **Conclusions:** This study underscores the transformative potential of ViTs in histopathological analysis, especially in resource-limited settings. By enhancing diagnostic accuracy and reducing dependence on high-quality imaging and specialized expertise, it presents a scalable solution for global cancer diagnostics. Future research should prioritize optimizing ViTs for such environments and broadening their clinical applications.

## 1. Introduction

Skin cancer, the most prevalent malignancy worldwide, originates from the uncontrolled growth of skin cells in the outermost layer of the body [1,2]. This disease has become alarmingly common, with approximately one in three cancer diagnoses being skin cancer and over 3.5 million cases reported each year in the United States [3,4]. Skin cancers can be broadly categorized into melanoma and nonmelanoma skin cancers (NMSCs), with the latter divided into basal cell carcinoma (BCC) and squamous cell carcinoma (SCC) [5]. While BCC generally exhibits a lower tendency for metastasis, SCC represents a significant health risk, contributing substantially to NMSC-related metastasis and mortality [6,7]. SCC, in particular, poses a greater clinical challenge owing to its potential for aggressive spread and distinct histological classifications, ranging from well-differentiated (Grade I) to poorly differentiated (Grades II and III) and undifferentiated or invasive stages (Grade IV) [8].

Primary SCC diagnosis often involves dermoscopic examination and tissue biopsy, followed by Mohs micrographic surgery (MMS), where tissue samples are microscopically analyzed to assess for residual cancer cells [9,10]. Effective margin assessment in SCC treatment is essential to ensure complete removal of tumor cells, which is crucial for reducing recurrence [11]. However, manual microscopic examinations demand substantial expertise and are heavily dependent on the judgment of trained pathologists [12,13]. The variability in diagnostic accuracy due to subjective interpretation has led to a demand for computational assistance through artificial intelligence (AI) and deep learning models for histopathological image analysis [14].

**Figure 1:**
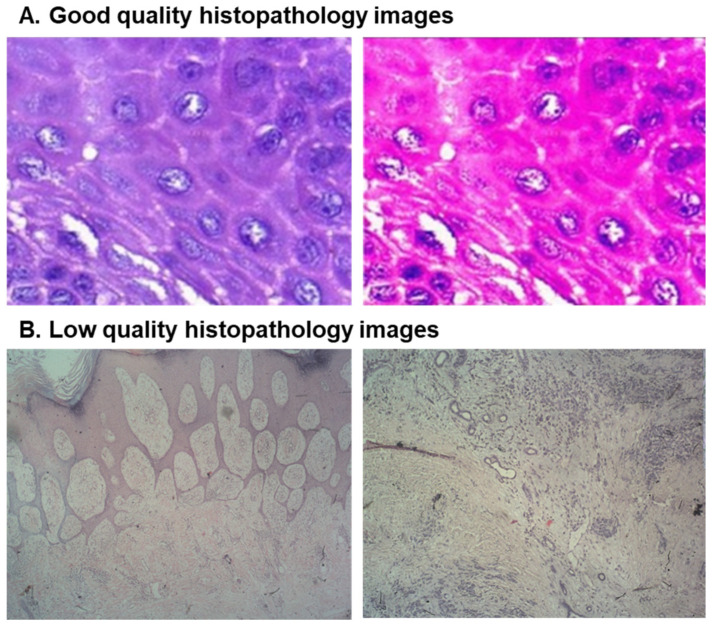
Comparison of quality of margin negative histopathological images with 2048 × 1536 pixels captured with 400× magnification from different settings. (**A**) Good quality histopathology image from a well-resourced setting. (**B**) Low-quality histopathology image from low-resource setting.

Histopathological image quality is critical in AI-based diagnostic accuracy [15] and is affected by multiple factors, including microscope quality, staining techniques, reagent reliability, skill level of laboratory personnel, and environmental conditions during diagnostic processes [16,17]. In resource-limited settings, the lack of high-quality equipment and trained personnel often results in low-quality histopathological images [18,19]. Figure 1 presents a comparative example. Figure 1A, from a well-resourced laboratory in India, demonstrates the clarity and detail that can aid in precise diagnosis [20]. In contrast, Figure 1B from a low-resource laboratory in Ethiopia showed limitations in image quality owing to substandard equipment and resources, which can impair accurate diagnosis and treatment planning [21].

Over the past decade, convolutional neural networks (CNNs) are the leading AI models for histopathological image analysis [22]. However, their performance is highly sensitive to image quality, leading to decreased accuracy when analyzing low-quality images [23,24,25]. Vision transformers (ViTs), an emerging architecture in deep learning, have recently gained popularity because of their ability to outperform CNNs in various image analysis tasks, particularly in suboptimal image quality situations [26,27,28]. ViTs can capture long-range dependencies and structural details more effectively, making them promising candidates for improving diagnostic accuracy, even with low-quality images [29,30].

In this study, we proposed a ViT-based model specifically designed for histopathological images with compromised quality, focusing on the classification of SCC margins. By examining the efficacy of ViTs in this context, we aimed to demonstrate their potential to enhance diagnostic accuracy and clinical decision-making in settings with limited resources.

### Related Works

To address low-quality histopathological image analysis, researchers have developed diverse methodologies aimed at enhancing image interpretability and diagnostic accuracy [31,32,33,34]. A common approach involves image enhancement techniques to improve visibility and standardization across various datasets [33]. Techniques such as stain normalization adjust color distributions within slides to counter inconsistencies in different staining protocols [33,35]. Noise reduction and contrast adjustment further improve the clarity of tissue details, making the images more suitable for machine learning interpretation [36,37]. These preprocessing steps mitigate common issues in low-resource settings, where imaging technology may be limited, outdated, or inconsistently calibrated [38,39].

Data augmentation and synthetic data generation address the limitations posed by small, low-quality datasets, which are common in under-resourced environments [40,41]. Data augmentation methods create variations in existing images by rotating, flipping, and scaling to increase model exposure to diverse image conditions [42]. Synthetic data generation techniques, such as generative adversarial networks (GANs), allow researchers to produce realistic histopathological images that resemble actual samples, including rare or less common cancer types [37,43]. These methods increase model robustness by providing additional training data, which is particularly beneficial for low-quality images with limited datasets [44].

Domain adaptation methods are also critical in adapting models trained on high-quality images to work effectively with low-quality images [45,46,47]. Techniques such as transfer learning leverage pretrained models and fine-tune them on smaller datasets from low-quality sources, reducing the need for large datasets [48]. Unsupervised domain adaptation methods were also explored, adjusting the model weights to better capture the features of low-resolution images from low-resource settings [49,50]. These approaches allow the cross-compatibility of diagnostic models across different imaging environments, helping bridge the quality gap without requiring additional training on low-quality data [39].

Patch-based analysis techniques, particularly those involving multiple instance learning (MIL), provide a localized approach for analyzing histopathological images of heterogeneous quality [51]. This method divides whole-slide images into smaller high-resolution patches, allowing the model to focus on specific tissue regions with clear features [52]. By aggregating the diagnostic information from each patch, patch-based methods can effectively reduce the impact of artifacts or blurred areas within low-quality slides [53]. Combined with hybrid models that integrate classical image processing techniques and CNNs, patch-based approaches have shown promise for achieving high diagnostic accuracy in low-quality imaging environments [54]. Collectively, these methodologies offer a comprehensive toolkit for improving AI-driven diagnostics in resource-constrained settings [38,39].

## 2. Materials and Methods

### 2.1. Dataset and Preprocessing

**Figure 2:**
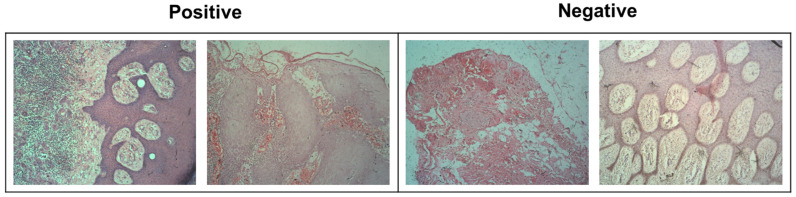
Positive and negative sample images (with 2048 × 1536 pixels captured with 400× magnification) of SCC margins.

Tissue samples were sourced from the Jimma University Medical Center, a leading healthcare facility in Ethiopia [21]. The dataset can be accessed publicly at (https://osf.io/3ma4p/) (accessed on 28 August 2024) and provides an important resource for researchers and clinicians. Tissue samples were obtained from patients diagnosed with skin-related conditions, specifically SCC. Hematoxylin and eosin (H&E) staining, a widely used method for the histological examination of tissue samples, was consistently applied across all samples. This standard staining method aids in highlighting cellular structures, allowing for easier visualization of abnormalities, such as cancerous cells and tissue margins. The dataset comprises histopathological slides obtained from 50 patients, offering a diverse representation of SCC grades. The distribution of patients included 17 with well-differentiated SCC, 15 with moderately differentiated SCC, and 18 with invasive SCC, reflecting varied degrees of cancer progression. This variation in differentiation stages is critical for training machine learning models that need to differentiate between varying levels of tumor aggressiveness. Additionally, the dataset included 345 normal tissue images that were classified as margin negative and represented healthy tissues. In contrast, 483 images containing tumor cells were designated as margin positive, indicating the presence of cancer at the tissue margins. The source dataset is particularly valuable for studying SCC margin classification, which is critical for ensuring complete tumor resection during surgery. Margin classification is crucial for determining whether cancer cells remain at surgical margins, which can influence treatment decisions and patient outcomes. By providing these images, along with their margin labels, the dataset supports the development of AI models aimed at automating the detection of tumor cells in low-quality or resource-limited settings, where manual analysis by pathologists may be challenging. Example images of normal and tumor tissue samples are shown in Figure 2.

The original resolution of the SCC margin cell images in this study was 2048 × 1536 pixels, which is a high level of detail typically required for histopathological analyses. However, to make the images more manageable and suitable for deep learning models, all images were resized to 224 × 224 pixels. This reduction in resolution is a common practice in machine learning to reduce the computational overhead while preserving key tissue features for analysis. A size of 224 × 224 pixels is often preferred for CNNs and other machine learning models because it offers a balance between processing efficiency and maintaining the necessary details for identifying abnormalities, such as cancerous cells. Furthermore, image resizing facilitates the generation of smaller, localized portions of the original images, enabling the model to focus on specific tissue regions and increase its ability to detect tumors even in lower-quality images. The patch-based approach is particularly useful in histopathology because tumors or abnormal cells may appear differently depending on their location within the tissue.

To further improve model performance and prevent overfitting, where the model becomes overly tuned to the training data, various data augmentation techniques were applied. These methods include flipping, scaling, and image rotation. Flipping the images horizontally or vertically mimicked the natural variations in tissue orientation during sample preparation and imaging. Scaling adjusts the size of the images, simulating cases in which the tumor or tissue features may appear larger or smaller, which is common in clinical practice. Rotation allows the model to learn from images at different angles, thereby improving its robustness to variations in slide presentation. These augmentations artificially increase the size and diversity of the training dataset, allowing the model to generalize better to unseen data, which is particularly critical when working with small datasets or images from resource-limited settings, where data variability can be high. By creating multiple versions of each image, the model learns more generalized patterns, preventing it from memorizing the specific features of the training images, thus improving its ability to accurately classify new images.

To assess the generalization capabilities of the model and further reduce the risk of overfitting, 5-fold cross-validation was used. This technique divides a dataset into five equally sized subsets or folds. The model was trained on four folds and tested on the remaining folds; this process was repeated for each fold. This ensured that every sample in the dataset was used for both training and testing, thereby providing a more robust evaluation of model performance. In this study, 90% of the dataset was used for training, allowing the model to learn from a large portion of the data, whereas the remaining 10% was used for testing. Cross-validation provides a more reliable estimate of the performance of the model on unseen data, ensuring that it does not overfit any particular subset of the data. In this work, we used stratified cross-validation to address the class imbalance in the dataset. Stratified cross-validation ensures that each fold of the dataset maintains the same proportion of classes as the original dataset, which is especially important in cases of imbalanced data. By preserving the distribution of classes across all folds, we ensured that the model is trained and evaluated on representative samples of both the majority (margin positive) and minority (margin negative) classes. This approach helps to mitigate the risk of biased performance metrics and allows the model to learn more effectively from the underrepresented class, ultimately leading to a more robust and fair evaluation of its performance.

### 2.2. Best Model and Parameters Selection

Preliminary experiments were conducted to identify the most suitable CNN and ViT models for the task, along with optimization of their key parameters. The initial selection included three CNN architectures, ResNet50, EfficientNetB2, and InceptionV3, and three ViT models, ViT-B16, ViT-B32, and ViT-L32. These models were evaluated using a range of learning rates (0.0001, 0.001, 0.01, and 0.1) and five optimizers: Adam, Adadelta, Adamax, Adagrad, and Stochastic Gradient Descent (SGD). The aim of this study was to explore how these factors influenced the ability of the model to classify SCC margin images effectively and to choose the best optimized parameters and models. In these experiments, the models’ pretrained ImageNet weights were retained, and only the final output layer was replaced and fine-tuned for the specific task. A fixed number of 50 epochs was selected for training to allow sufficient learning while maintaining computational efficiency.

**Figure 3:**
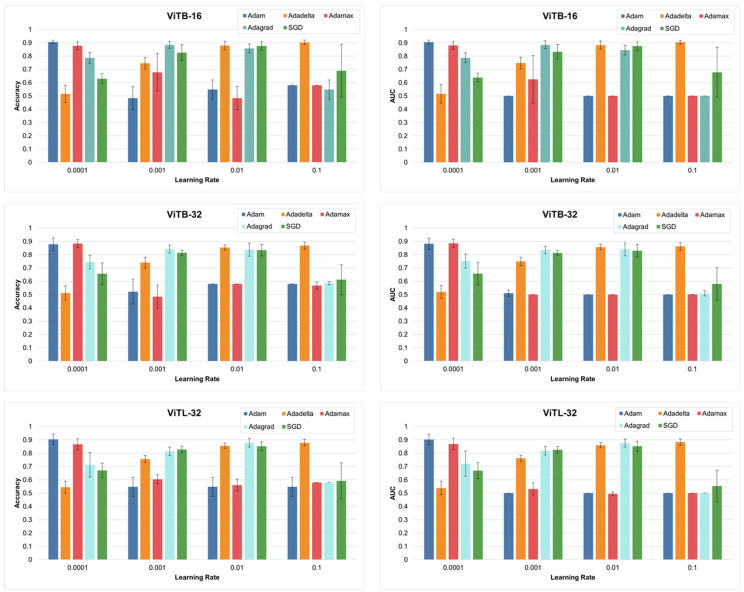
Preliminary study results of pretrained vision transformers on SCC images to select best model and parameter combination.

The results of these preliminary experiments, which guided the selection of the best-performing models, are shown in Figure 3. In these early tests, the performance differences between the models and optimizers were assessed based on the classification accuracy and area under the receiver operator curve (AUC) values. The outcomes revealed valuable insights into the behavior of the models under different configurations, helping to refine the choice of architecture and optimization strategy for subsequent experiments. From the preliminary experiments, we identified the best-performing model for this study. The ViT model, ViT-B16, was selected as the optimal ViT, whereas the InceptionV3 model was chosen as the best CNN model. These models were selected based on their superior performance in terms of classification accuracy and efficiency when applied to the dataset used in this study. In addition to selecting the models, optimal training parameters were determined. Among the various optimizers tested, the Adam optimizer yielded the best results for both the ViT and CNN models. The ability of Adam to adaptively adjust the learning rate during training makes it particularly effective for fine-tuning models on a specific histopathology dataset. Moreover, a learning rate of 0.0001 emerged as the most effective starting point for both models because it balanced the speed of convergence with stable training and minimized the risk of overfitting. Therefore, based on the results of these preliminary experiments, the study proceeded with the ViT-B16 and InceptionV3 models using the Adam optimizer with a starting learning rate of 0.0001 as the final selected parameter for further training and evaluation.

### 2.3. Proposed Model

In this study, a ViT-based transfer learning approach was used to classify low-quality SCC images as either positive (present tumor) or negative (no tumor). The core idea behind transfer learning is to leverage a model pretrained on a large, generalized dataset, such as ImageNet, and fine-tune it for a specific task, in this case, the classification of histopathological images. Pretraining on ImageNet allows the model to learn the general features of images, such as edges, textures, and basic shapes, which can then be adapted for more specialized applications, such as SCC image classification. This approach is particularly beneficial when working with limited data as it reduces the need for extensive training from scratch and low-quality images and adds to the previous knowledge acquired from large datasets such as ImageNet.

**Figure 4:**
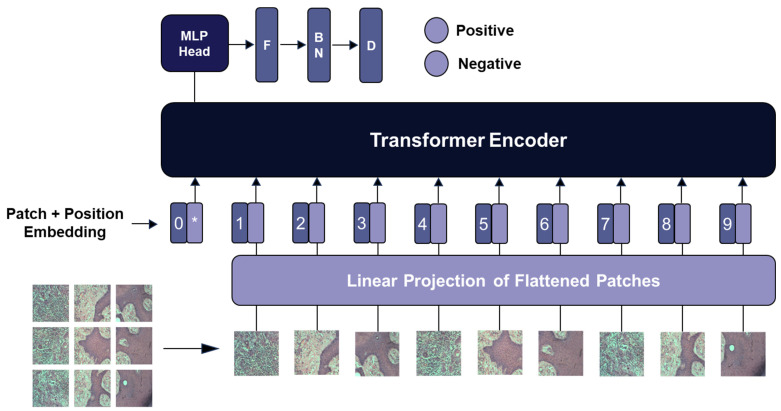
Structure of proposed ViT-based model for low-quality SCC image classification. *, extra learnable (class) embedding; 0, …, 9, patch location number.

The transfer learning model in this study is based on ViT, a deep learning architecture that has gained attention owing to its success in handling image classification tasks. The ViT model, originally pretrained on the ImageNet dataset, was customized for SCC image classification by modifying the architecture after the Multi-Layer Perceptron (MLP) head. Specifically, after the pretrained ViT layers, a flattening layer was added to convert the 2D features into a 1D vector, followed by a batch normalization layer to stabilize learning and prevent overfitting. In the final step, dense layers were added to allow the model to learn complex relationships within the data, followed by a SoftMax output layer to classify the images into two categories: margin negative (normal) and margin positive (tumor present). The architecture and design of the model, including additional layers, are shown in Figure 4.

### 2.4. Implementation

The best model was trained at a learning rate of 0.0001 and was selected based on preliminary experiments to ensure stable convergence. The training was performed on a high-performance computing (HPC) cluster with NVIDIA GPUs (NVIDIA Corporation, Santa Clara, CA, USA), enabling the efficient processing of the computationally intensive ViT model and a large dataset of SCC images. The training was performed over 200 epochs, a duration found to be effective for capturing the intricate details of histopathological structures without overfitting. The model weights were optimized using the Adam optimizer, which was selected for its adaptability and efficiency in deep learning tasks. A batch size of 64 was used to balance the memory efficiency and training speed. Additionally, L2 regularization was applied in the form of weight decay, which aided in preventing the model from learning overly complex patterns that could lead to overfitting.

### 2.5. Performance Measures

In this study, several performance metrics were employed to evaluate the ability of the model to classify SCC margin images accurately. The values were assessed using five-fold cross-validation with a 95% confidence interval. These metrics included accuracy, precision, recall, F1 score, and AUC. Each metric provides a unique perspective on model performance, offering insights into how well the model balances between identifying true positives and minimizing false positives. For *TP*, true positive; *TN*, true negative; *FP*, false positive; *FN*, false negative, the metrics are given in Equations (1)–(4) below.

(1) $$\mathrm{Accuracy}=\frac{TP+TN}{TP+TN+FP+FN}$$

(2) $$\mathrm{Precision}=\frac{TP}{TP+FP}$$

(3) $$\mathrm{Recall}=\frac{TP}{TP+FN}$$

(4) $$F1=2\times \frac{Precision\times Recall}{Precision+Recall}$$

## 3. Results

To evaluate the proposed ViT-based approach for low-quality histopathological images in classifying SCC cases, we ran experiments involving different epochs, employing addition and removal layers, and compared them with the best-performing CNN models. Consequently, the proposed ViT-based transfer learning approach performed better than the CNNs and various additional layer combinations.

Table 1 presents the results of measuring the performance of the proposed method in terms of accuracy, AUC, F1 score, precision, and recall for various epoch numbers, including 50, 100, 150, 200, and 250 epochs. Among all epoch numbers utilized, the best model provided the highest performance results when running for 200 epoch, providing the most optimized performance. The proposed ViT-based SCC images classification provided the highest accuracy of 0.928 ± 0.027, AUC of 0.927 ± 0.028, F1 score of 0.926 ± 0.029, precision of 0.922 ± 0.028, and recall of 0.928 ± 0.027 running for 200 epochs.

**Figure 5:**
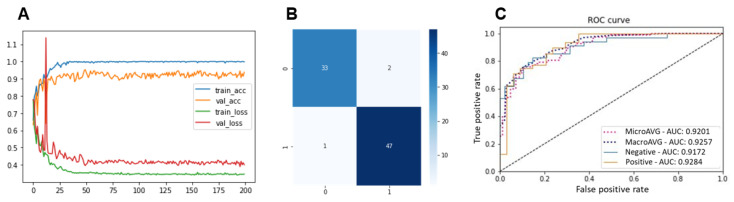
Visual machine learning performance outputs of proposed method. (**A**) Learning curve. (**B**) Confusion matrix. (**C**) ROC curve.

Figure 5 shows the learning curve (the *Y*-axis represents accuracy, and the *X*-axis represents the number of epochs), confusion matrix, and ROC curve of the proposed model. From the figure, it can be deduced that the learning regimen was smooth, making the proposed model optimal for classifying SCC image data. The training, validation, and test accuracies were 0.998 ± 0.002, 0.937 ± 0.010, and 0.928 ± 0.027, respectively. The training, validation, and test losses were 0.346 ± 0.015, 0.389 ± 0.008, and 0.390 ± 0.002, respectively. The confusion matrix shows a small number of misclassified images, proving the effectiveness of the proposed model in classifying SCC images that were never observed during training. The ROC curve showed an almost perfect result, demonstrating the capability of the proposed method for low-quality histopathological image classification.

The proposed ViT-based method was compared with CNN-based methods to evaluate the effectiveness of the ViT models in classifying low-quality histopathology images relative to CNNs. To do this, the three best-performing CNN models chosen from preliminary studies were selected, trained, and evaluated on the same data distribution as that of the ViT-based model by keeping all parameters the same as the parameters chosen from the preliminary study and were all optimized parameters for both the ViT and CNN models. The results of these comparisons are presented in Table 2. Based on these results, the highest performing CNN-based approach for low-quality histopathology image classification on the SCC dataset was InceptionV3 with the highest accuracy of 0.860 ± 0.049, AUC of 0.837 ± 0.029, F1 score of 0.854 ± 0.044, precision of 0.858 ± 0.057, and recall of 0.854 ± 0.039 running for 200 epochs. When compared to the ViT-based model that has the highest accuracy of 0.928 ± 0.027, AUC of 0.927 ± 0.028, F1 score of 0.926 ± 0.029, precision of 0.922 ± 0.028, and recall of 0.928 ± 0.027 running for 200 epochs, the ViT-based approach performed better than that of the CNN-based approach.

**Figure 6:**
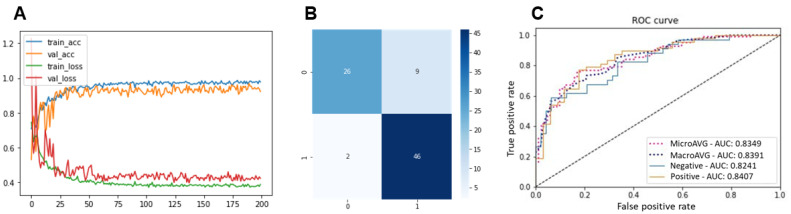
Visual machine learning performance outputs of best-performing CNN-based approach for SCC image classification (InceptionV3). (**A**) Learning curve. (**B**) Confusion matrix. (**C**) ROC curve.

A comparison of the CNN-based approach was also performed using visual machine learning measures, including the learning curve, confusion matrix, and ROC curve. Figure 6 shows the learning curve, confusion matrix, and ROC curve of the InceptionV3-based model (the highest performing CNN-based model) for classifying SCC images. Compared to the ViT-based approach depicted in Figure 5, the ViT-based approach provided better results when evaluated visually.

Furthermore, an ablation study involving the use of different layer configurations was conducted to evaluate whether this affected the performance of the SCC image classification. To this end, we designed a ViT-based approach with three configurations, including the first configuration with pretrained ViT with only the output layer attached; configuration two with a pretrained ViT model with a flattening layer, batch normalization, dense layer, batch normalization, and output layer; and the third configuration with a pretrained ViT model with a flattening layer, batch normalization, two dense layers, batch normalization, and output layer, which were used for comparison with the proposed model. As shown in Table 3, the proposed model outperformed all three configurations, although all three configurations provided better results than the best-performing CNN model, reinforcing the fact that ViT-based models outperformed CNN-based models in the classification of SCC images, irrespective of how the ViT-based approach was configured.

**Figure 7:**
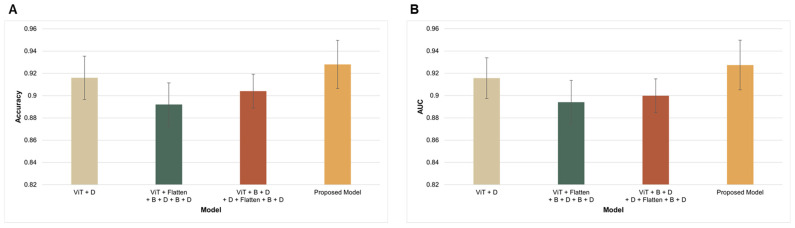
Comparison of different configurations of ViT-based transfer learning for SCC image classification in terms of (**A**) accuracy and (**B**) AUC.

Figure 7 shows the differences in performance (in terms of accuracy (Figure 7A) and AUC (Figure 7B)) between the various ViT-based configurations employed for the ablation study, reinforcing the fact that the proposed combination is the most optimized and effective architecture of the ViT-based approach for low-quality histopathological image classification.

## 4. Discussion

These findings highlight the potential of ViT-based models for addressing critical diagnostic challenges in histopathological analysis. Traditional CNN-based models are the benchmark for image classification tasks but are notably sensitive to image quality, limiting their efficacy in resource-constrained settings. By leveraging the global attention mechanism of ViTs, this study achieved a significant leap in the diagnostic accuracy of SCC margin classification, particularly with low-quality images.

A standout aspect of the ViT model is its ability to capture long-range dependencies and structural features, which are often blurred or degraded in suboptimal images. This capability is crucial for SCC margin classification, in which the fine details of tissue morphology are vital for accurate diagnosis. Additionally, the proposed architecture, which incorporates flattening, batch normalization, and dense layers, demonstrates the importance of tailored configurations for specific tasks. The ablation study reinforced the idea that even if modifications in the ViT architecture were made, they did not result in substantial performance variations. This indicates that the ViT-based approach is highly effective for low-quality histopathological images.

In addition to these technical achievements, this study has profound implications for clinical practice in under-resourced environments. By reducing the reliance on high-quality imaging equipment and pathologist expertise, the proposed model bridges the gap between technological capabilities and clinical needs, enabling more equitable access to effective cancer diagnostics. Moreover, the results emphasized the potential of AI to enhance diagnostic precision while reducing variability among pathologists. This aligns with the broader goals of precision medicine, in which personalized and accurate treatment decisions are paramount. Therefore, collaboration between healthcare providers and policymakers is essential for scaling AI-driven diagnostics in global health systems. In general, this work not only contributes to the body of research on transformer models in medical imaging but also provides a foundation for future studies aimed at addressing the challenges of diagnostic accuracy in low-quality imaging conditions.

However, the computational demands of ViTs pose a barrier to their widespread adoption, particularly in low-resource settings lacking high-performance hardware. For instance, compared to EfficientNetB2, ViT is computationally expensive having 80 M trainable parameters (against 5 M for EfficientNetB2), requiring longer training time of 5 h over 50 epochs (against 3 h for EfficentNetB2), and having GFLOPs of 17 (against 2 for EfficientNetB2). Future studies should explore lightweight adaptations of ViT models or hybrid architectures that balance computational efficiency with diagnostic accuracy. Expanding the dataset to include diverse SCC subtypes and integrating real-world clinical workflows will further validate the utility of this model. The evaluation focused on a single SCC dataset, which restricted the generalizability of the results to other histopathological image types and cancers. In addition, although the model successfully classified low-quality images, further investigation is required to assess its performance under varying clinical imaging conditions. The black-box nature of transformers also presents a challenge for clinical adoption because their decision-making processes are non-interpretable. This limitation could impact the clinicians’ ability to trust and utilize these models in practice.

Future studies should focus on expanding the dataset to include diverse types of histopathological images to assess the generalizability of the model across various cancers and imaging settings. Further research on interpretability methods for transformers would also enhance clinical transparency and trust in these models. Hybrid architectures that integrate the strengths of CNNs and transformers can offer another pathway for improving the performance and interpretability of complex visual recognition tasks in medical imaging. Furthermore, the issue of class imbalance in the dataset must be resolved by considering patient-level data split for cross-validation and considering more evaluation approaches such as using precision–recall curves and others.

## 5. Conclusions

This study established the efficacy of a ViT-based model for classifying SCC margins in low-quality histopathological images, thus outperforming CNN-based approaches in terms of accuracy, precision, and robustness. These results underscore the transformative potential of ViTs in addressing the diagnostic challenges inherent in resource-constrained settings where suboptimal imaging conditions often prevail. Our findings have several important implications. The proposed ViT model enhances diagnostic accuracy and democratizes access to reliable cancer diagnostics. By mitigating the dependency on high-quality imaging and specialized expertise, this approach has the potential to improve surgical outcomes and reduce global cancer recurrence rates. However, this study has some limitations. The computational requirements of ViTs remain a significant hurdle, particularly in low-resource environments where access to high-performance hardware is limited. Although diverse, the dataset used in this study may not capture the full spectrum of SCC presentations observed in clinical practice. Expanding the dataset and validating the model across various patient populations and imaging conditions will strengthen its clinical applicability. Future studies should focus on the development of lightweight and hardware-efficient ViT variants for real-time deployment. Furthermore, integrating this model into existing clinical workflows and evaluating its impact on patient outcomes is critical. The exploration of hybrid AI models that combine the strengths of ViTs and CNNs could also provide valuable insights. Ultimately, this study paves the way for advancing AI-driven histopathological analysis, with implications extending beyond SCC to other cancers and diseases and offering a scalable solution to global healthcare challenges.

## Figures and Tables

**Table 1:** Performance of proposed ViT-based model over different epochs.

Model	Epoch	Accuracy (95%)	AUC (95%)	F1 Score (95%)	Precision (95%)	Recall (95%)
ViTB-16	50	0.892 ± 0.014	0.895 ± 0.020	0.892 ± 0.014	0.892 ± 0.014	0.894 ± 0.023
	100	0.916 ± 0.019	0.916 ± 0.017	0.914 ± 0.019	0.910 ± 0.012	0.918 ± 0.016
	150	0.900 ± 0.015	0.903 ± 0.011	0.898 ± 0.010	0.898 ± 0.010	0.906 ± 0.014
	200	0.928 ± 0.027	0.927 ± 0.028	0.926 ± 0.029	0.922 ± 0.028	0.928 ± 0.027
	250	0.914 ± 0.031	0.905 ± 0.039	0.906 ± 0.034	0.916 ± 0.027	0.904 ± 0.039

**Table 2:** Comparison of proposed ViT-based approach against CNNs-based approach.

Model	Accuracy (95%)	AUC (95%)	F1 Score (95%)	Precision (95%)	Recall (95%)
ResNet50	0.766 ± 0.024	0.755 ± 0.033	0.724 ± 0.044	0.726 ± 0.030	0.734 ± 0.037
EfficientNetB2	0.734 ± 0.040	0.745 ± 0.044	0.734 ± 0.030	0.736 ± 0.032	0.738 ± 0.022
InceptionV3	0.860 ± 0.049	0.837 ± 0.029	0.854 ± 0.044	0.858 ± 0.057	0.854 ± 0.039
Proposed Model	0.928 ± 0.027	0.927 ± 0.028	0.926 ± 0.029	0.922 ± 0.028	0.928 ± 0.027

**Table 3:** Ablation study results using different additional layer configurations.

Model	Configuration	Accuracy (95%)	AUC (95%)	F1 Score (95%)	Precision (95%)	Recall (95%)
ViTB-16	ViT + D	0.916 ± 0.024	0.916 ± 0.023	0.916 ± 0.024	0.916 ± 0.020	0.916 ± 0.024
	ViT + Flatten + B + D + B + D	0.892 ± 0.024	0.894 ± 0.024	0.890 ± 0.025	0.894 ± 0.024	0.890 ± 0.025
	ViT + B + D + D + Flatten + B + D	0.904 ± 0.019	0.900 ± 0.012	0.900 ± 0.012	0.904 ± 0.019	0.900 ± 0.012
	Proposed model	0.928 ± 0.027	0.927 ± 0.028	0.926 ± 0.029	0.922 ± 0.028	0.928 ± 0.027

B, batch normalization; D, dense layer.

## Data Availability

In this study, histopathological images obtained from the Jimma University Medical Center were used (data are publicly available and can be accessed at https://osf.io/3ma4p/ (accessed on 28 August 2024)).
